# *Geijigajakyak* decoction inhibits the motility and tumorigenesis of colorectal cancer cells

**DOI:** 10.1186/s12906-016-1281-z

**Published:** 2016-08-15

**Authors:** Soong-in Lee, Jeong A Bae, Yoo-Seung Ko, Kyoung-in Lee, Hangun Kim, Kyung Keun Kim

**Affiliations:** 1 Medical Research Center for Gene Regulation, Chonnam National University Medical School, Kwangju, Korea; 2 College of Korean Medicine, DongShin University, Naju, Korea; 3 Biotechnology Industrialization Center, DongShin University, Naju, Korea; 4 College of Pharmacy, Sunchon National University, Sunchon, Korea; 5 Department of Pharmacology, Chonnam National University Medical School, Hak-Dong 5, Dong-Ku, Kwangju, 61469 Korea

**Keywords:** Colorectal cancer, Herbal medicine, Geijigajakyak decoction, Cell invasion, Tumorigenesis

## Abstract

**Background:**

Recent studies report that inflammatory diseases of the large intestine are associated with colorectal cancer. *Geijigajakyak* Decoction (GJD) has antispasmodic and anti-inflammatory effects on the gastrointestinal tract. Thus, in light of the connection between chronic bowel inflammation and colorectal cancer (CRC), we asked whether GJD inhibits colorectal tumorigenesis.

**Methods:**

The effects of GJD on the viability and proliferation of CRC cells were evaluated using MTT and BrdU assays, respectively. The motility of CRC cells was examined by a Transwell migration/invasion assay and immunoblot analysis was used to examine the signaling pathways associated with migration. A syngeneic Balb/c mice allograft model, in which CT26 cells were injected into the dorsum, was used to evaluate the anti-tumor effects of GJD in vivo.

**Results:**

GJD had no cytotoxic effects against HCT116 CRC cells, although it did inhibit their proliferation. GJD inhibited the migration of HCT116 cells, and suppressed the invasion of HCT116, Caco2, and CSC221 CRC cells. In addition, GJD downregulated the expression of p-JNK and p-p38 MAPK, which are downstream signaling molecules associated with invasiveness. Furthermore, oral administration of GJD (333 mg/kg, twice a day) inhibited tumor growth in a mouse xenograft model.

**Conclusions:**

GJD inhibited the motility of human CRC cells and suppressed tumorigenesis in a mouse model. These results suggest that GJD warrants further study as a potential adjuvant anti-cancer therapy.

**Electronic supplementary material:**

The online version of this article (doi:10.1186/s12906-016-1281-z) contains supplementary material, which is available to authorized users.

## Background

Complementary and Alternative Medicine (CAM) is popular with cancer patients today. CAM therapies are not a main treatment for cancer, but they can be used as adjuncts to conventional therapies such as radiotherapy, chemotherapy, hormone therapy, and surgery [1, 2]. Herbal formulae have a long history as a form of CAM therapy, and have engendered strong trust among those that practice and receive Korean traditional medicine. Today, some herbal formulations are thought to affect multiple pharmacological targets; as such, they are expected to be a useful component of combination therapies that show better efficacy and greater safety than single compound-based drugs [3].

The *Shanghanlun*, an ancient Chinese medical report, introduces the *Geijigajakyak* Decoction (GJD; Gui Zhi Jia Shao Yao Tang) in the section dealing with greater yin disease, which covers all diseases with symptoms such as abdominal fullness, food accumulation, diarrhea, and abdominal pain [4]. If patients with greater yin disease experience abdominal fullness and pain, GJD is prescribed as the optimal drug; indeed, it is used to treat many gastrointestinal diseases, including colitis. Recent studies report that GJD reduces abdominal pain by altering intestinal movement [5], and has significant anti-inflammatory effects in rats with 2,4,6-trinitrobenzene sulfonic acid-induced colitis by inhibiting smooth muscle contraction and neutrophil chemotaxis [6]. Other studies report that GJD has antispasmodic and antidepressant effects in those with irritable bowel syndrome [7], that it has antidiarrheal effects [8], and that it relaxes gastrointestinal smooth muscle [9, 10]. However, no study has examined the effects of GJD on gastrointestinal cancer.

There are 1.2 million cases of colorectal cancer (CRC) per year worldwide, with 600,000 deaths. Indeed, CRC is the third most common cancer worldwide, and metastasis is the major cause of death. The 5-year survival rate for patients with distant metastasis at the time of diagnosis is 0–7 % [11]. Up-regulation of cancer cell motility is an essential step in metastasis and tumor progression [12]; indeed, metastasis is the main cause of death in about 90 % of human cancer cases. Thus, inhibiting cancer cell migration and invasion may suppress metastasis. We previously studied the effects of modulating gene expression on progression of colorectal tumorigenesis via examining cell motility and signaling in vitro and measuring tumor growth in vivo in a syngeneic mouse model [13, 14].

Many studies suggest a strong correlation between colorectal tumorigenesis and chronic bowel inflammation [15–17], and several herbal prescriptions used to treat gastrointestinal symptoms have been tested to see whether they have any anti-cancer effects; for example, PHY906 has been tested as a modulator of chemotherapy [18], as an adjuvant therapy for cancer [19], as a modulator of irinotecan-based therapy [20], and as an attenuator of chemotherapy-induced gastrointestinal toxicity [21]. *Shaoyao* Decoction (SYD), another herbal prescription, improves colitis-associated CRC [22]. As GJD might function as a complementary agent to alleviate chronic bowel inflammation, and in light of the connection between chronic inflammation and CRC, we thus asked in this study whether GJD suppresses CRC similar to PHY906 and SYD. Therefore, we investigated the effects of GJD on colorectal tumorigenesis by examining cell motility and signaling in vitro, and its effects in a syngeneic mouse tumor model. We found that GJD inhibited the motility of CRC cells in vitro and colorectal tumorigenesis in vivo.

## Methods

### Preparation of GJD

GJD comprises five commonly used herbs: Cinnamomi Ramulus, Glycyrrhizae Radix, Paeoniae Radix, Zingiberis Rhizoma, and Ziziphi Fructus. The raw herbs used to prepare GJD were purchased from Omniherb (Additional file 1: Table S1, Daegu, Korea) and mixed at a ratio of 3:6:2:3:3; the weight of each herb (gram, dry weight) is 18, 36, 12, 18, and 18 g, respectively (Table 1). Aqueous extract of GJD was prepared by suspending the herb mixture (total 102 g) in 1 l of distilled water and heating to 100 °C for 3 h in a water bath (KSB-55; Sunil Developed ENG, CO., LTD., Korea). Aqueous extract of Paeoniae Radix (PE) was also prepared by suspending the herb (100 g, dry weight) in 1 l of distilled water with the same method as GJD. The extracts were then filtered through filter paper (Whatman™ Cat No. 1004 150; GE Healthcare, UK) and concentrated using a vacuum evaporator (R124; Buchi Labortechnik AG, Switzerland). Finally, they were lyophilized by freeze-drying (FD 8508; Ilshin Lab, CO., Ltd. Korea) and stored at −20 °C. GJD powder was diluted in water prior to use. After dilution, the solution was filtered through a 45 Ø filter and stored at −20 °C. When added to culture media, the final volume of GJD solution was limited to less than 5 % to prevent osmotic shock.

**Table 1:** Ingredients and doses of *Geijigajakyak* Decoction (GJD)

Herb	Known antioxidants and phenolic compounds	References	Weight (g)	
			Used to prepare sample	One day administration of decoction (in the *Shanghanlun*)
Cinnamomi Ramulus	Phenolic acids (cinnamic acid, protocatechuric acid), coumarin, tannins, eugenol, 2-hydroxycinnamaldehyde	[2, 33–35]	18	9
Glycyrrhizae Radix	Flavanones (dihydroflavones: liquiritin, liquirigenin), licopyranocoumarins, 18β-glycyrrhetinic acid, isoliquiritigenin	[2, 36–38]	36	18
Paeoniae Radix	Flavonols (astragalin), tannins (gallotannin), stilbenes (resveratrol), adenosine, betulinic acid, oleanolic acid, paeoniflorin, paeonol, α-tocopherol	[2, 25, 39–44]	12	6
Zingiberis Rhizoma	Phenolic volatile oils (gingerol analogues: gingerols, shogaols)	[2, 45–50]	18	9
Ziziphi Fructus			18	9

### Cell culture

Caco2, HCT116, and CT26 (colorectal cancer) cell lines were purchased from the American Type Culture Collection (ATCC, Manassas, VA) and CSC221 (a colorectal cancer) cell line was purchased from the BioMedicure (San Diego, CA). HCT116, Caco2, CSC221, and the CT26 murine colon cancer cell line were maintained at 37 °C in a 5 % CO_2_ atmosphere in DMEM supplemented with 10 % FBS and 1 % penicillin/streptomycin. All cell lines used in the study were authenticated by the ATCC and BioMedicure using STR-PCR analysis.

### Cell viability assay

Cell viability was measured in a 3-(4, 5-dimethylthiazol-2-yl)-2, 5-diphenyltetrazolium bromide (MTT) assay using the EZ-Cytox Cell viability assay kit (Daeil Lab Service Co., Korea). Cells were treated with GJD and seeded in 96-well flat bottomed plates at a density of 1 × 10^4^ cells/100 ml. The culture medium was removed after 24 or 48 h. Next, 10 μl of EZ-Cytox reagent and GJD-treated medium was added to each well for another 4 h at 37 °C prior to measurement of cell viability. The absorbance was determined in an ELISA micro-plate reader at a test wavelength of 450 nm.

### Cell proliferation assay

Cell proliferation was measured according to the level of 5-bromo-2-deoxyuridine (BrdU) incorporation during DNA synthesis. The assay was performed using the Cell proliferation ELISA BrdU kit according to the manufacturer’s protocol (Roche, Mannheim, Germany). In brief, 1 × 10^4^ cells were incubated with 100 μl of test compound (0–1.0 mg/ml GJD) in 96-well flat bottomed plates for 24 or 48 h. Cells were then treated with BrdU labeling solution for 2 h. The culture medium was then removed, the cells fixed, and DNA denatured. Cells were incubated with Anti-BrdU-POD solution for 90 mins and antibody conjugates were removed through three washing cycles. Immune complexes were detected by incubation with a TMB substrate for 15 min and quantified by measuring the absorbance at 390 nm and 472 nm. All tests were performed in duplicate, with six wells per treatment group. All experiments were repeated at least twice.

### Cell migration and invasion assays

Cell migration and invasion were measured using a Transwell apparatus as described previously [13]. Briefly, to measure cell invasion, the top chamber of each well of a 24 well Transwell chamber was coated overnight at 37 °C with 1 % gelatin. Wells were not coated with gelatin when measuring cell migration. After incubation, the gelatin solution was removed from the upper chamber, which was then allowed to dry for 4 h. Medium (500 μl), containing fibronectin as a chemoattractant, was then added to the bottom chamber of each well. Cells (1 × 10^5^ or 2 × 10 in DMEM/0.2 % BSA) were seeded in the upper chamber and incubated at 37 °C for 24 or 48 h. After incubation, the cells were stained and examined under a microscope (Leica Microsystems).

### Western blot analysis

Western blot analysis was performed to examine the cell signaling events affected by GJD. After treatment with GJD, cells were lysed in RIPA Lysis buffer (25 mM Tris · HCl, pH 7.6, 150 mM NaCl, 1 % NP-40, 1 % sodium deoxycholate, 0.1 % SDS) containing a protease inhibitor cocktail (Sigma). Lysates were then incubated for 30 min on ice, followed by centrifugation for 10 min. The protein concentration in the supernatants was measured using a BCA protein assay reagent (Bio-Rad). Aliquots were loaded onto SDS-electrophoresis gels, separated, and transferred to a PVDF membrane. The membrane was then immunoblotted with antibodies specific for Akt (Cell Signaling), p-Akt (Cell Signaling), c-Jun (Cell Signaling), ERK (Cell Signaling), p-ERK (Cell Signaling), p-JNK (Cell Signaling), p-p38 MAPK (Cell Signaling), and β-actin (Santa Cruz), followed by secondary antibodies conjugated to horseradish peroxidase (Amersham). Reactive bands were visualized by enhanced chemiluminescence using a LAS 3000 (Fuji Film, Tokyo, Japan).

### In vivo tumor growth

The CT26 cell/syngeneic mouse model was used to investigate the in vivo effects of GJD on colorectal tumorigenesis, as it is reported that a syngeneic mouse tumor model is a good for testing the anti-cancer effects of candidate substances in short-term studies [11]. Male Balb/c mice (5 weeks old) were purchased from DaMul Science, Korea, and acclimated for 1 week prior to subcutaneous injection of syngeneic CT-26 cells (2 × 10^5^) into the dorsum as previously described [14]. After 7 days, tumors were palpable and mice were randomly assigned to vehicle (PBS)-treated or GJD-treated groups (*n* = 7 mice/group). GJD (333 mg/kg; dose calculated to maintain a serum concentration of 1.0 mg/ml) was orally administered twice per day; control mice received PBS. The time line of the protocol is outlined in Fig. 5a. The experimental protocol was approved by the Chonnam National University Medical School Research Institutional Animal Care & Use Committee, and animals were maintained and all experiments performed according to the Guiding Principles in the Care and Use of Animals (DHEW publication, NIH 80-23). Tumor volume ($V$) was calculated using the following equation: $V$ = 1/2×$a$×$b$^2^, where $a$ and $b$ are the longest and shortest diameters of the tumor (in millimeters), respectively. Tumor volume was measured daily for 21 days to verify the effects of GJD. All mice were sacrificed after Day 21, and the subcutaneous tumor grafts were surgically excised and weighed.

### Liquid chromatography mass spectrometry (LC-MS) analysis

Aqueous extract of GJD and aqueous extract of Paeoniae Radix (PE) samples were analyzed using an Agilent LC-1200 series instrument combined with an Agilent 6410 triple-quadrupole mass spectrometer (Agilent Technologies, USA) system. A YMC-Pack Pro C8 column (4.6 × 150 mm, 3 μm, YMC, Japan) was coupled to the system and the flow rate was set at 0.7 mL/min. The mobile phases comprised 5 mM ammonium acetate in water containing 0.1 % formic acid (A) and 5 mM ammonium acetate in methanol containing 0.1 % formic acid (B). The gradient was programmed as follows: 0–1 min, 70 % A; 1–5 min, 70–20 % A; 5–8 min, 20–5 % A; 8–13 min, 5 % A; 13–14 min, 5–70 % A; 14–25 min, 70 % A. The injection volume was 5 μl. Mass spectrometry analysis was performed in the multiple reaction monitoring with negative-ion electrospray ionization (ESI-) mode. The fragment electric voltage, collision energy, and quantification of paeonol were achieved by monitoring the *m/z* of precursor/product ions (Table 2). Synthesized paeonol compound (Aldrich) was used as a standard for calibration.

**Table 2:** Mass spectrometry parameters used for paeonol analysis

Compound	Precursor/product ions (*m/z*)	Fragment electric voltage (V)	Collision energy (eV)
Paeonol	165/150, 122	80	10

### Statistical analysis

Experimental differences were tested for statistical significance using ANOVA followed by Tukey’s HSD post-hoc test or Student’s *t* test. All statistical tests were two-sided and *P*-values <0.05 were considered significant. Statistical analysis was performed using PASW Statistics 20 (SPSS) software.

## Results

### GJD shows a mild anti-proliferative effect against CRC cells

**Fig. 1:**
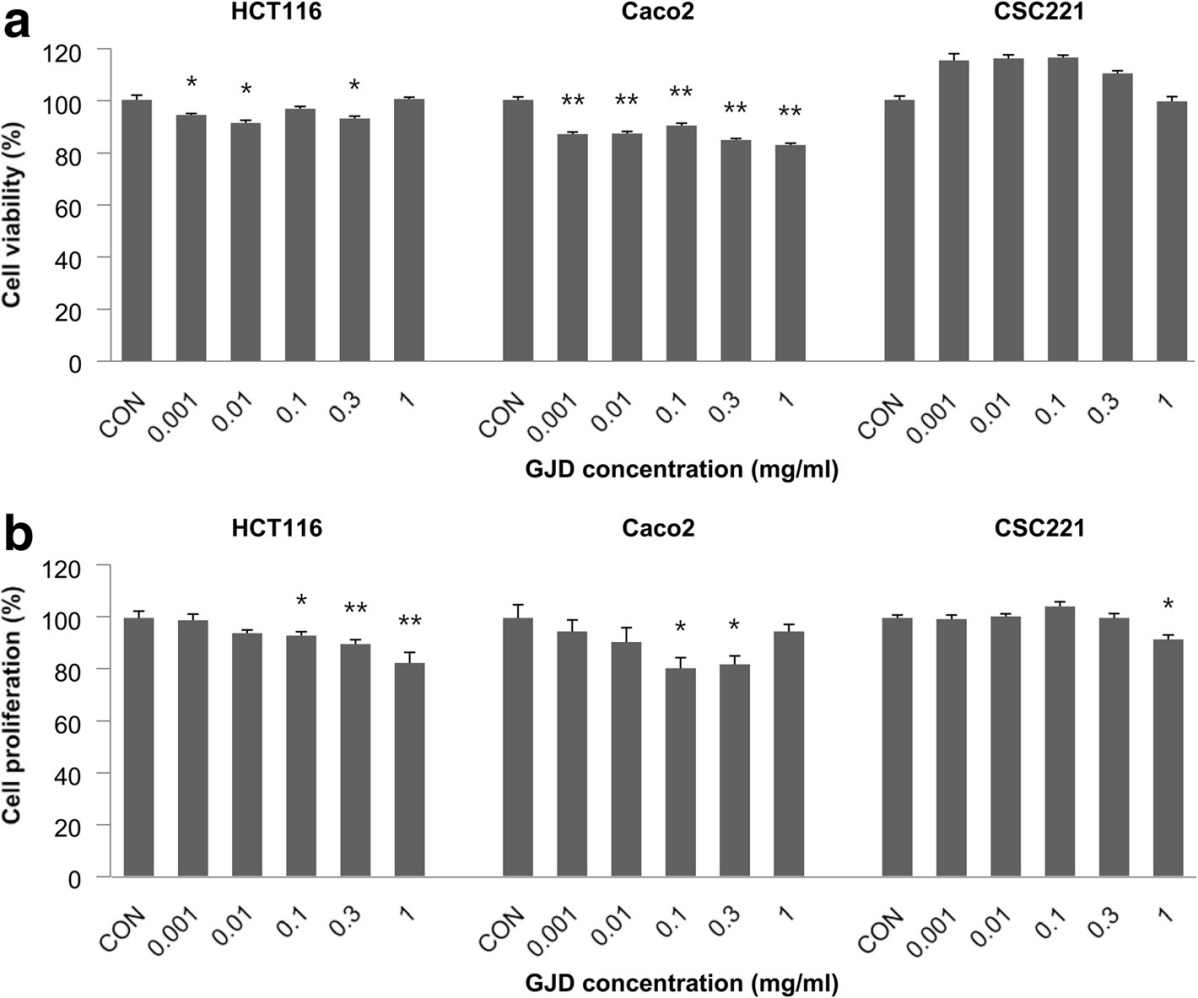
GJD shows mild anti-proliferative effects against human colorectal cancer cells. **a** Cell viability was measured in an MTT assay and compared with that of control (CON) cells. Each bar represents the mean ± SEM for triplicate samples. An *asterisk* indicates a significant difference between cells treated with GJD and vehicle (PBS) (*P < 0.05, **P < 0.01). HCT116 cells (1 × 10^4^ cells/well) were incubated with 0.001–1 mg/ml GJD for 48 h. Caco2 and CSC221 cells (1 × 10^4^ cells/well) were incubated with 0.001–1 mg/ml GJD for 24 h. **b** Cancer cell proliferation was measured in a BrdU assay and compared with that of control cells

The effects of GJD on the viability of CRC cells were measured in MTT assays. As shown in Fig. 1a, HCT116, Caco2, and CSC221 cells showed differing viabilities in the presence of increasing doses of GJD. The viability of HCT116 cells cultured for 48 h in the presence of 0.3 mg/ml GJD was 93.05 % of that of untreated controls. By contrast, the viability of Caco2 and CSC221 cells in the presence of 0.3 mg/ml GJD for 24 h was 84.75 and 110.25 %, respectively, of that of untreated controls. These results indicate that GJD was only very mildly toxic at a concentration of 0.3 mg/ml.

As shown in Fig. 1b, the proliferation of HCT116 cells showed a dose-dependent decrease upon exposure to GJD. At a concentration of 0.3 mg/ml, GJD suppressed HCT116 proliferation to 89.81 % of that of untreated controls. However, the proliferation of other cells showed an inconsistent pattern. At 0.3 mg/ml, GJD suppressed the proliferation of Caco2 cells to 81.96 % of that of untreated controls. By contrast, the proliferation of CSC221 cells was unaffected at GJD doses from 0.001 mg/ml to 0.3 mg/ml. These results indicate that GJD has only a mild anti-proliferative effect on human CRC cells. When CRC cell viability and proliferation rates were compared at a concentration of 0.3 mg/ml GJD, we recorded values of 93.05 % vs. 89.81 %, 84.75 % vs. 81.96 %, and 110.25 % vs. 99.93 % for HCT116, Caco2, and CSC221 cells, respectively, indicating that GJD at 0.3 mg/ml suppresses cell proliferation a little better than cell viability. In other words, although GJD is not toxic to these cells, it does suppress their proliferation. Thus, a concentration of 0.3 mg/ml GJD was selected as the optimal dose for studying its anti-invasive effects against HCT116, Caco2, and CSC221 cells.

### GJD inhibits the migration and invasion of colorectal cancer cells

**Fig. 2:**
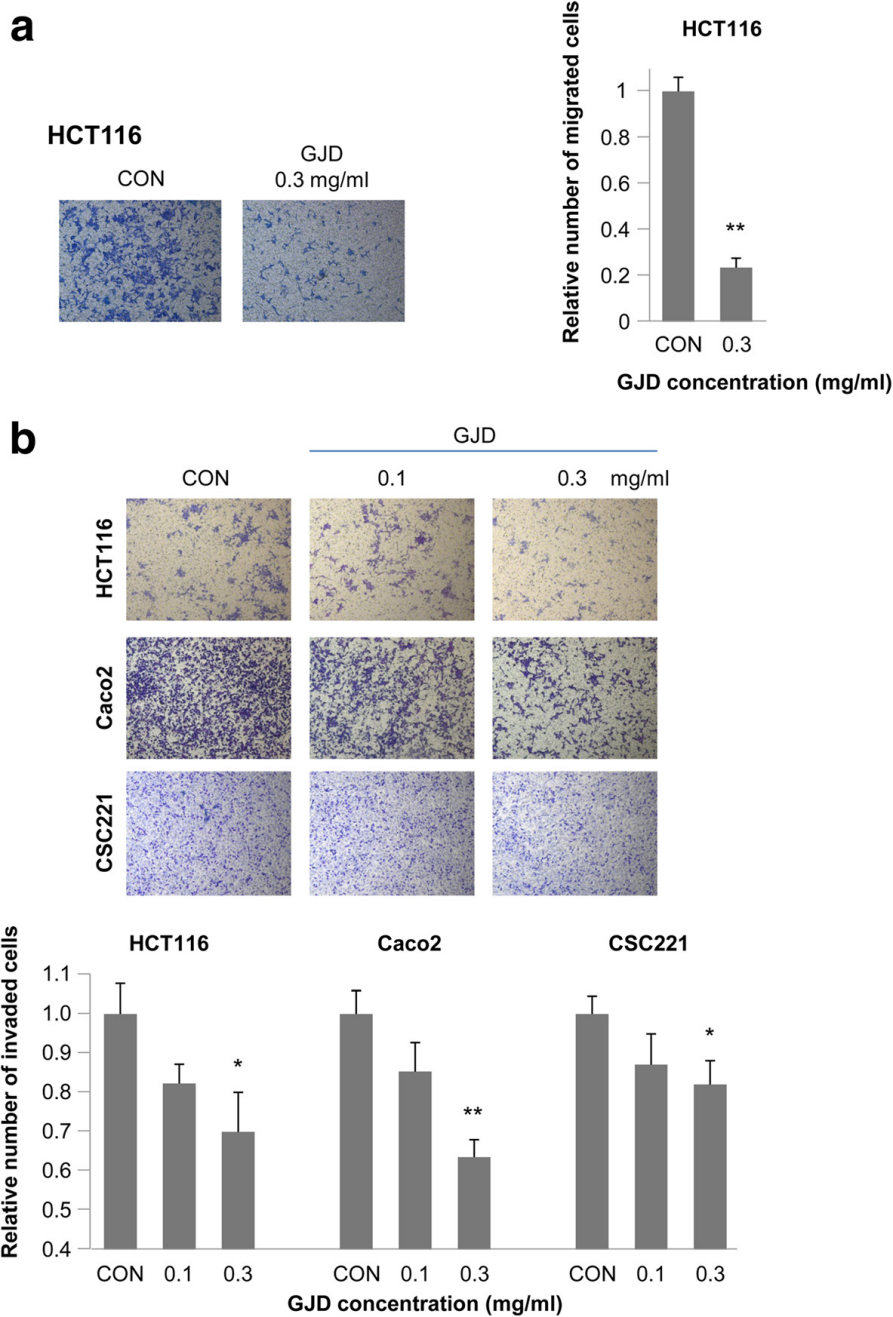
GJD inhibits the motility of colorectal cancer cells. **a** GJD suppresses the migration of colorectal cancer cells. HCT116 cells were treated with different concentrations of GJD (0.1 or 0.3 mg/ml) or with vehicle (PBS), and subjected to a migration assay: upper wells, 1 × 10^5^ cells and 0.2 % BSA; lower wells, 1 % FBS and 10 μl/ml fibronectin. After 48 h, cells were stained and analyzed. The images shown are representative of three independent experiments. The histogram represents the number of migrating cells, which was counted in five chosen areas (mean ± SEM, *n* = 3). An *asterisk* indicates a significant difference between the GJD and control groups (**P < 0.01). **b** GJD suppresses the invasion of colorectal cancer cells. HCT116, Caco2, and CSC221 cells were treated with different concentrations of GJD (0.1 or 0.3 mg/ml) or vehicle (PBS) and subjected to an invasion assay. HCT116 cells were incubated for 48 h: upper wells, 1 × 10^5^ cells and 0.2 % BSA; lower wells, 1 % FBS and 10 μl/ml fibronectin. Caco2 and CSC221 cells were incubated for 24 h: upper wells, 1 × 10^5^ cells and 0.2 % BSA; lower wells, serum free medium and 10 μl/ml fibronectin. The histogram represents the number of invading cells, which was counted in five chosen areas (mean ± SEM, *n* = 3). An *asterisk* indicates a significant difference between the GJD and control groups (*P < 0.05, **P < 0.01)

A Transwell migration assay was used to evaluate whether GJD affects the migration/invasion of HCT116 cells. HCT116 migration was 23.49 % of that of untreated control cells in the presence of 0.3 mg/ml GJD, indicating that GJD inhibits CRC migration. The results of the invasion assays based on HCT116, Caco2, and CSC221 cells showed that invasion decreased as the concentration of GJD increased, i.e., the effect was dose-dependent (Fig. 2a). As shown in Fig. 2b, GJD significantly inhibited the invasion of HCT116, Caco2, and CSC221 cells at 0.3 mg/ml. Invasion of HCT116, Caco2, and CSC221 cells in the presence of 0.3 mg/ml GJD was 69.91, 63.50, and 85.78 %, respectively, of that of untreated control cells. As shown above, the viability of HCT116, Caco2, and CSC221 cells in the presence of 0.3 mg/ml GJD was 93.05, 84.75, and 110.25 %, respectively, of that of untreated controls. Thus GJD inhibited cell invasion regardless of cell viability.

### Effect of GJD on the downstream signaling events involved in HCT116 cell invasion

**Fig. 3:**
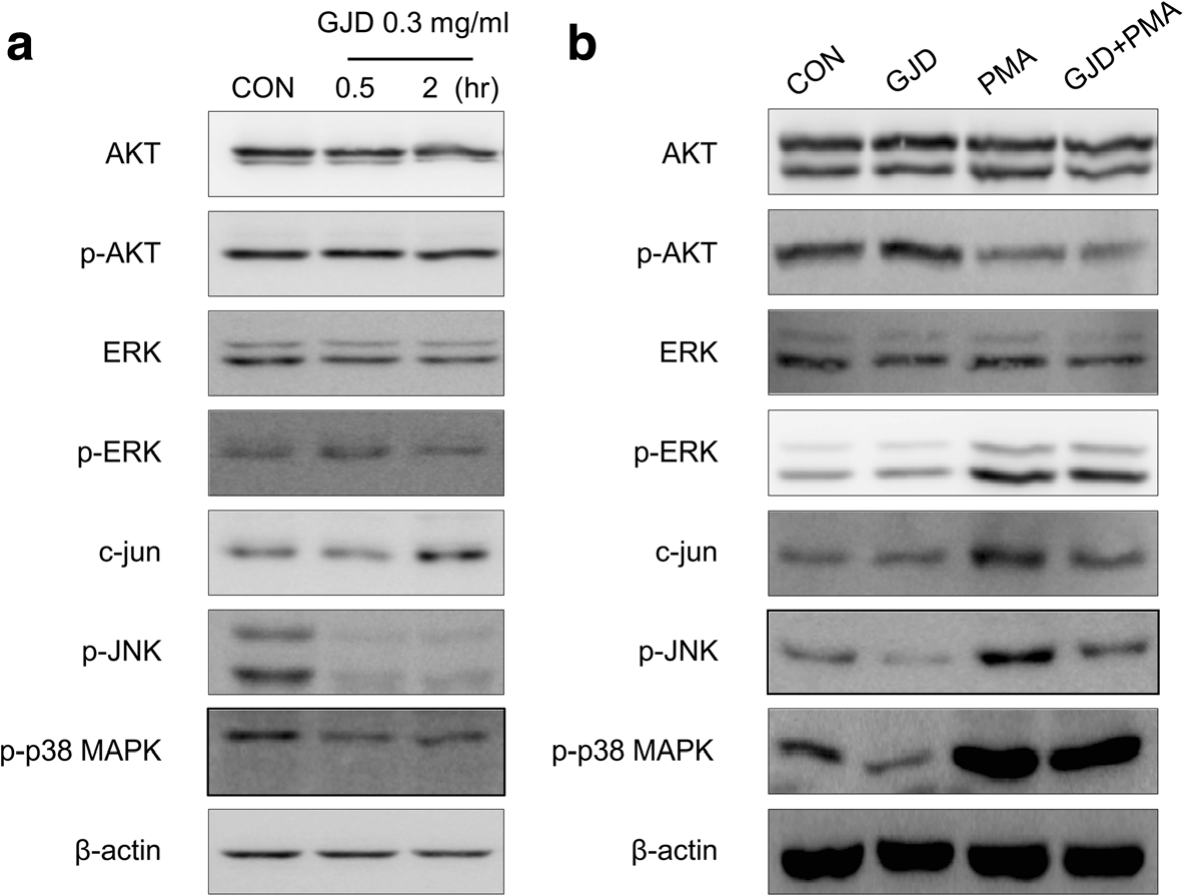
Effects of GJD on the expression of p-JNK and p-38 MAPK in HCT116 cells. **a** HCT116 cells were treated with 0.3 mg/ml GJD for different times (0, 0.5, or 2 h), after which Akt, p-Akt, ERK1/2, p-ERK1/2, c-Jun, p-c-Jun, p-JNK, and p-p38 levels were examined by western blotting. **b** HCT116 cells were pretreated with 0.3 mg/ml GJD for 1 h before exposure to 100 nM PMA for 15 min. Whole cell lysates were then subjected to immunoblot analysis as in (**a**)

Next, we performed western blot analysis to examine the cell signaling pathways responsible for the anti-invasive phenotype conferred upon CRC cells by GJD. Changes in the expression of Akt, p-Akt, ERK, p-ERK, c-jun, p-JNK, and p-p38 were examined at 30 min and 2 h post-treatment of HCT116 cells with 0.3 mg/ml GJD. As shown in Fig. 3a, expression of p-JNK and p-p38 MAPK decreased after exposure to GJD. It is well-known that phorbol-myristic acid (PMA) activates protein kinase C isozymes, which are upstream regulators of the MAP kinase pathways, to promote tumor progression [23]. Therefore, we stimulated HCT116 with PMA to identify the signaling molecules that play a role in the inhibitory effects of GJD. HCT116 cells were treated with GJD for 1 h and then activated with PMA for 15 min. As shown in Fig. 3b, GJD downregulated PMA-induced expression of p-JNK, c-Jun, and p-p38. Thus, the inhibitory effect of GJD is related to its ability to inhibit the phosphorylation of JNK and p-38 MAPK.

### GJD suppresses the proliferation and motility of CT26 murine colon cancer cells

**Fig. 4:**
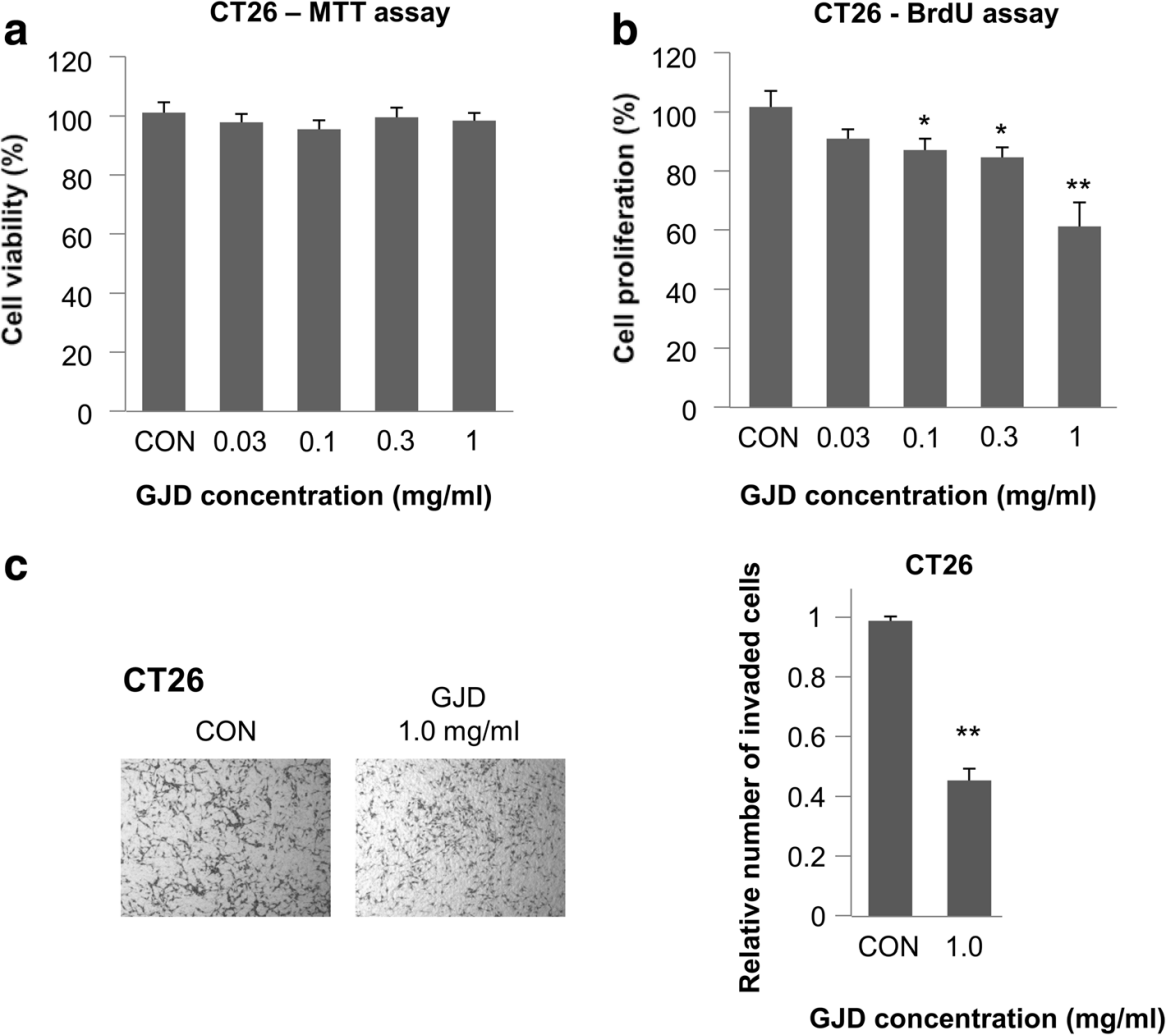
GJD is not cytotoxic to CT26 cells, but does suppress their proliferation and invasion. **a** Effects of GJD on the viability of CT-26 cells. CT26 cells (1 × 10^4^ cells/well) were incubated with 0–1.0 mg/ml GJD for 24 h and cell viability was measured in an MTT assay. **b** Effect of GJD on the proliferation of CT-26 cells. CT-26 cells (1 × 10^4^ cells/well) were incubated with 0–1.0 mg/ml GJD for 24 h and proliferation was measured in a BrdU assay. **c** Effect of GJD on the invasion of CT-26 cells. CT-26 cells were incubated for 24 h in the presence of vehicle or 1.0 mg/ml GJD and subjected to an invasion assay: upper wells, 1 × 10^5^ CT-26 cells and 0.2 % BSA; lower wells: serum free medium and fibronectin (10 μl/ml). The histogram represents the number of invading cells, which was counted in five chosen areas (mean ± SEM, *n* = 3). An *asterisk* indicates a significant difference between the GJD and control groups (**P < 0.01)

CT26 cells were the first choice for studying the effect of GJD on colorectal tumorigenesis in vivo because they are a murine colon adenocarcinoma cell line that is tumorigenic in Balb/c mice. CT26 viability was measured in an MTT assay in the presence of different concentrations of GJD. GJD at concentrations up to 1 mg/ml had no effect on cell viability (97.29 % of that of untreated controls; Fig. 4a). Therefore, a concentration of 1.0 mg/ml was selected for further studies.

Proliferation of CT26 cells in the presence of different concentrations of GJD was measured in a BrdU assay. In contrast to cell viability, GJD reduced cell proliferation in a dose-dependent manner. As shown in Fig. 4b, the proliferation of CT26 cells was reduced to 60.13 % of that of controls in the presence of 1.0 mg/ml GJD. These results indicate that although GJD is not cytotoxic at 1.0 mg/ml, it does inhibit the proliferation of CT26 cells.

Transwell invasion assays revealed that GJD significantly inhibited the invasion of CT26 cells at a concentration of 1.0 mg/ml. As shown in Fig. 4c, invasion was 46.12 % of that of untreated control cells. This result indicates that GJD inhibited the invasion of CT26 cells regardless of cell viability.

### GJD suppresses tumor growth in a syngeneic mouse xenograft model

**Fig. 5:**
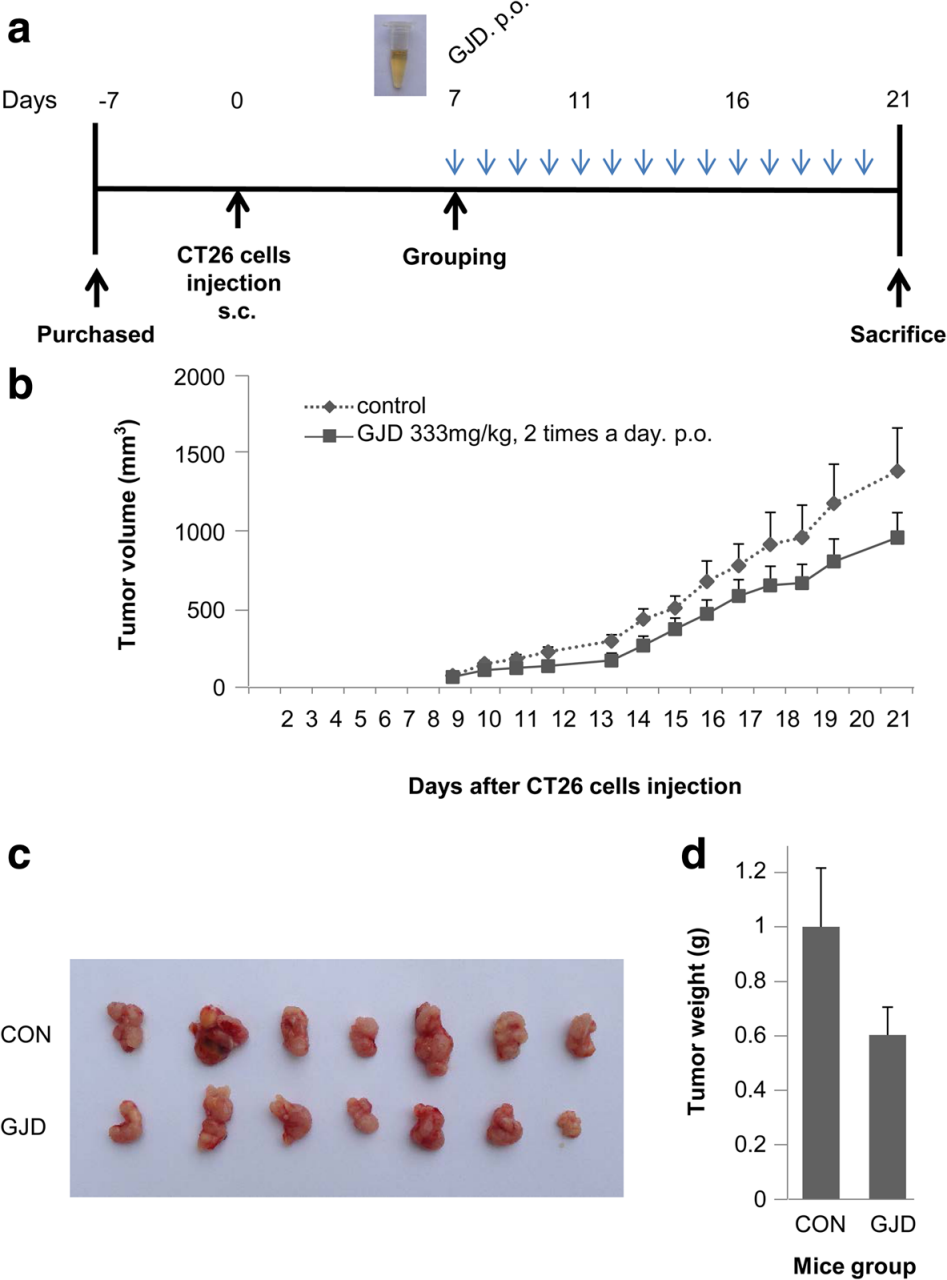
GJD inhibits tumor growth in a syngeneic mouse tumor model. **a** Time line for the generation of a mouse tumor xenograft model and the schedule for GJD treatment. Vehicle (PBS) was orally administered to control mice twice a day. Test mice received 333 mg/kg GJD twice a day. **b** Effect of GJD on tumor formation by CT-26 cells. Tumor volumes, measured daily and calculated using the formula width^2^ × length × 0.5 (length > width), are expressed as the mean ± SEM. Tumors in the GJD group (*n* = 7) grew more slowly than those in the control group (*n* = 7). **c** Photographs of tumor tissues isolated from each group. **d** Tumor weights were measured in each group at Day 21. Data are expressed as the mean ± SEM. GJD inhibited tumor formation by CT-26 cells

Finally, we examined the effects of GJD on tumor growth in a mouse tumor xenograft model. Seven days after subcutaneous injection of CT26 cells (2 × 10^5^) into the dorsum of Balb/c mice, animals were treated with either vehicle (PBS) or GJD. GJD-treated mice received 333 mg/kg twice a day, while control group mice were orally administered with PBS twice a day (Fig. 5a). Tumor progression was then compared between groups; the results showed that and GJD inhibited tumor progression (Fig. 5b, c). The average tumor volume in the control group at Days 7 and 21 was 61.92 mm^3^ and 1380.76 mm^3^, respectively, while that in the GJD group was 53.95 mm^3^ and 954.08 mm^3^, respectively. As shown in Fig. 5d, the average tumor weight in the control group was 1.00 g, whereas that in the GJD group was 0.60 g, although the difference did not reach statistical significance.

### Paeonol is not detected in the aqueous extracts of GJD

**Fig. 6:**
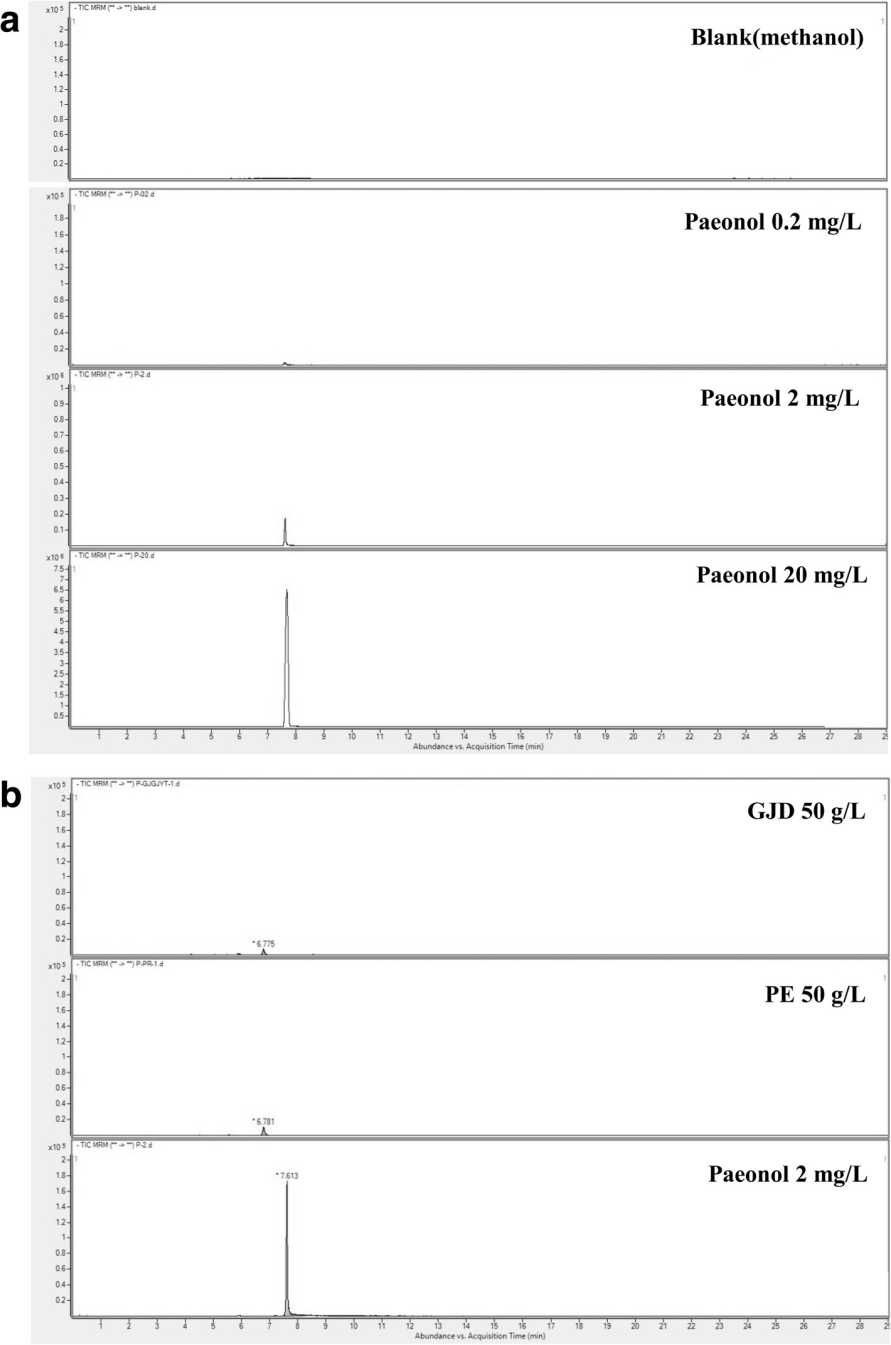
LC-MS chromatograms to detect paeonol in aqueous extract of GJD. The MS analysis was performed in the multiple reaction monitoring with negative-ion electrospray ionization mode and the transition of *m*/*z* 165 → 150, 122 for paeonol. **a** Blank (methanol; solvent) and paeonol standard (0.2, 2, 20 μg/ml). **b** Aqueous extracts of PE (50 mg/ml) and GJD (50 mg/ml). Paeonol was not detected in the PE and GJD extracts

GJD comprises Cinnamomi Ramulus, Glycyrrhizae Radix, Paeoniae Radix, Zingiberis Rhizoma, and Ziziphi Fructus (Table 1). These five herbs contain phenolic acids, coumarins, tannins, flavanones, flavonols, stilbenes, and phenolic volatile oils, which may have anti-cancer properties [2]. In particular, paeonol from Paeoniae Radix has anti-inflammatory and anti-oxidative [24] and anti-angiogenic and anti-metastatic [25] activities, and inhibits melanoma metastasis [26]. Thus, we chose paeonol as one of candidates contributing GJD’s anti-tumor activity and asked whether paeonol might contribute to the anti-invasive effects of GJD on CRC cells observed in this study. To do this, we examined chromatograms of GJD by LC-MS with multiple reaction monitoring mode analysis (from 165 *m/z* to 150 *m/z* and 122 *m/z*) (Fig. 6). Synthesized paeonol compound was used as a standard for calibration (Fig. 6a). As paeonol is contained in Paeoniae Radix [24–26] among the five herbs (Table 1), aqueous extract of Paeoniae Radix (PE) was included as another control for detecting paeonol. However, paeonol was not detected in aqueous PE and GJD extracts even in the concentration of 50 mg/ml, which is about 50 times of concentration used in in vitro and in vivo study (Fig. 6b). Therefore, it appears that the observed anti-tumor activity of GJD against CRC cells might not be wholly dependent on paeonol.

## Discussion

Many studies have attempted to identify the anti-cancer effect of various decoctions from traditional Chinese medical formulations; for example. the effects of Guizhi-Fuling-decoction on cervical cancer [27], Lichong decoction on uterine leiomyoma [28], Kuan-Sin-Yin decoction on bladder and lung cancers [29], ShaoYao decoction on colitis-associated colorectal cancer [22], Shu-Gan-Liang-Xue decoction on breast cancer [30], and Jiedu Xiaozheng Yin decoction [31] and Songyou Yin decoction [32] on hepatoma. Cell motility assays and examination of downstream signaling pathways have been used to elucidate the mechanisms underlying their action in vitro. Also, tumor-bearing mice models were developed to assess the ability of decoctions to inhibit tumor growth in vivo. These decoctions suppress tumor growth both in in vitro and in vivo [27–32].

GJD contains many components that affect cell motility, such as cinnamic acid [33], eugenol [34], and 2-hydroxycinnamaldehyde [35] from Cinnamomi Ramulus; 18β-glycyrrhetinic acid [36] and isoliquiritigenin [37, 38] from Glycyrrhizae Radix; adenosine [39], betulinic acid [40], oleanolic acid [41, 42], paeoniflorin [43], paeonol [25], and α-tocopherol [44] from Paeoniae Radix; gingerol [45–47], and 6-shogaol [48–50] from Zingiberis Rhizoma. Among these components, we chose paeonol, which was reported to have anti-tumor activity [25, 26], as one of candidates to contribute to the anti-invasive effects of GJD on CRC cells. We thus examined chromatograms of aqueous extracts of GJD by LC-MS, but we did not detect paeonol in the GJD used in this study. When considering that most phenolic compounds affecting cell motility have very limited water solubility and GJD is aqueous extract, we suppose that GJD does not depend on the paeonol for its anti-tumor activity against CRC cells. However, other unknown constituents of GJD may mediate the effects against CRC cells observed in this study and further studies should identify the components of GJD that confer anti-invasive properties.

Metastasis is the main cause of death in CRC patients. Metastasis comprises many steps; however, inhibition of cell signaling may be a useful therapy [51]. Here, we found that GJD (0.3 mg/ml) markedly inhibited the invasion of HCT116, Caco2, and CSC221 CRC cells. Moreover, GJD suppressed expression of p-JNK and p-p38 MAPK. Abnormal MAPK signaling plays a critical role in cancer progression [52]. Down-regulation of p-JNK and p-p38 MAPK by GJD may lead to inhibition of HCT116 invasion. These results are similar to those reported in an advanced study of the ability of GuaLou-GuiZhi Decoction to inhibit LPS-induced microglial cell motility by interfering with the MAPK signaling pathway [53]. p38 MAPK modulates cancer cell invasion and migration; thus, interfering with this signaling pathway may inhibit tumor metastasis [54]. Therefore, these results indicate that GJD inhibits the cell signaling pathways associated with invasion, regardless of cell viability. In this study, we observed that oral administration of 333 mg/kg GJD twice a day inhibited tumorigenesis in Balb/c mice. The dose was calculated to yield a blood concentration of 1 mg/ml based on a body weight of 30 g. A previous study showed that cinnamic acid, hippuric acid, paeoniflorin, and glycyrrhetic acid, all of which are components of Guizhi decoction (the same herbal composition but different ratios to GJD), have half-lives ranging from 1.2 ± 0.3 h to 6.6 ± 2.5 h in rats [55]. Thus, three or more oral doses per day may be appropriate; however, we gave the drug twice a day to minimize the stress to the mice. Taken together, our present results showed that GJD via oral administration could delay colorectal tumor progression.

Our present results and other reports provide some speculation that GJD may be a useful adjuvant therapy for CRC. First, the relationship between tumorigenesis and inflammation has been examined in many studies; these studies provide much genetic, pharmacological, and epidemiological evidence to support such a link; also, inflammatory bowel disease is a critical factor for the progression of colon cancer [15–17]. Second, immune cells and pro-inflammatory cytokines play important roles during the development of inflammation-induced cancers; conversely, colon tumors can induce inflammation of the colon [56]. Third, inflammatory mediators and components of the tumor microenvironment influence metastatic events [57]. Thus, considering the connection between chronic bowel inflammation and CRC, GJD act similarly to other decoctions reported to inhibit colorectal tumorigenesis, e.g., PHY906 [18–21] and SYD [22]. Taken all together, the data reported herein suggest that GJD may be an effective adjuvant therapy for CRC.

## Conclusions

Here, we show that GJD inhibits the motility of human CRC cells and suppresses tumorigenesis in a mouse model. Also, GJD inhibits the p-JNK and p-p38 MAPK cell signaling pathways, which are associated with invasion, regardless of cell viability. Although further studies should identify the components that endow GJD with anti-invasive properties, the present results suggest that GJD may be a potential adjuvant anti-cancer therapy for CRC.

## Additional file

Additional file 1: Table S1. Herbal information of five components of GJD used in this study. (DOCX 15 kb)

## Abbreviations

BrdU: 5-bromo-2-deoxyuridine
CAM: Complementary and Alternative Medicine
CRC: Colorectal cancer
GJD: Geijigajakyak decoction
LC-MS: Liquid chromatography mass spectrometry
MTT: 3-(4, 5-dimethylthiazol-2-yl)-2, 5-diphenyltetrazolium bromide
PBS: Phosphate buffered saline
PE: Aqueous extract of paeoniae radix
PMA: Phorbol-myristic acid
SYD: *Shaoyao* decoction

## References

[1] Adams M, Jewell AP (2007). The use of complementary and alternative medicine by cancer patients. Int Semin Surg Oncol.

[2] Cai Y, Luo Q, Sun M, Corke H (2004). Antioxidant activity and phenolic compounds of 112 traditional Chinese medicinal plants associated with anticancer. Life Sci.

[3] Schmidt BM, Ribnicky DM, Lipsky PE, Raskin I (2007). Revisiting the ancient concept of botanical therapeutics. Nat Chem Biol.

[4] Lee SI (2014). A philological research on the Way of medical study of ShangHanLun. Herbal Formula Sci.

[5] Iizuka N, Hamamoto Y (2015). Constipation and herbal medicine. Front Pharmacol.

[6] Lee JY, Kang HS, Park BE, Moon HJ, Sim SS, Kim CJ (2009). Inhibitory effects of Geijigajakyak-Tang on trinitrobenzene sulfonic acid-induced colitis. J Ethnopharmacol.

[7] Oka T, Okumi H, Nishida S, Ito T, Morikiyo S, Kimura Y, Murakami M (2014). Effects of kampo on functional gastrointestinal disorders. Biopsychosoc Med.

[8] Saitoh K, Kase Y, Ishige A, Komatsu Y, Sasaki H, Shibahara N (1999). Effects of Keishi-ka-shakuyaku-to (Gui-Zhi-Jia-Shao-Yao-Tang) on diarrhea and small intestinal movement. Biol Pharm Bull.

[9] Nobe K, Momose K, Sakai Y (2002). Effects of kampo medicine, keishi-ka shakuyaku-to (TJ-60) on alteration of diacylglycerol metabolism in gastrointestinal smooth muscle of diabetic rats. Acta Pharmacol Sin.

[10] Kito Y, Teramoto N (2012). Effects of Hange-shashin-to (TJ-14) and Keishi-ka-shakuyaku-to (TJ-60) on contractile activity of circular smooth muscle of the rat distal colon. Am J Physiol Gastrointest Liver Physiol.

[11] Arnold D, Seufferlein T (2010). Targeted treatments in colorectal cancer: state of the art and future perspectives. Gut.

[12] Hanahan D, Weinberg RA (2000). The hallmarks of cancer. Cell.

[13] Lee JH, Park SR, Chay KO, Seo YW, Kook H, Ahn KY (2004). KAI1 COOH-terminal interacting tetraspanin (KITENIN), a member of the tetraspanin family, interacts with KAI1, a tumor metastasis suppressor, and enhances metastasis of cancer. Cancer Res.

[14] Lee JH, Cho ES, Kim MY, Seo YW, Kho DH, Chung IJ (2005). Suppression of progression and metastasis of established colon tumors in mice by intravenous delivery of short interfering RNA targeting KITENIN, a metastasis-enhancing protein. Cancer Res.

[15] Terzic J, Grivennikov S, Karin E, Karin M (2010). Inflammation and colon cancer. Gastroenterology.

[16] Ekbom A, Helmick C, Zack M, Adami HO (1990). Ulcerative colitis and colorectal cancer. A population-based study. N Engl J Med.

[17] Eaden JA, Abrams KR, Mayberry JF (2001). The risk of colorectal cancer in ulcerative colitis: a meta-analysis. Gut.

[18] Farrell MP, Kummar S (2003). Phase I/IIA randomized study of PHY906, a novel herbal agent, as a modulator of chemotherapy in patients with advanced colorectal cancer. Clin Colorectal Cancer.

[19] Liu SH, Cheng YC (2012). Old formula, new Rx: the journey of PHY906 as cancer adjuvant therapy. J Ethnopharmacol.

[20] Kummar S, Copur MS, Rose M, Wadler S, Stephenson J, O’Rourke M, Brenckman W, Tilton R, Liu SH, Jiang Z (2011). A phase I study of the chinese herbal medicine PHY906 as a modulator of irinotecan-based chemotherapy in patients with advanced colorectal cancer. Clin Colorectal Cancer.

[21] Lam W, Bussom S, Guan F, Jiang Z, Zhang W, Gullen EA, Liu SH, Cheng YC (2010). The four-herb Chinese medicine PHY906 reduces chemotherapy-induced gastrointestinal toxicity. Sci Transl Med.

[22] Lin X, Yi Z, Diao J, Shao M, Zhao L, Cai H, Fan Q, Yao X, Sun X (2014). ShaoYao decoction ameliorates colitis-associated colorectal cancer by downregulating proinflammatory cytokines and promoting epithelial-mesenchymal transition. J Transl Med.

[23] Stapleton CM, Joo JH, Kim YS, Liao G, Panettieri RA, Jetten AM (2010). Induction of ANGPTL4 expression in human airway smooth muscle cells by PMA through activation of PKC and MAPK pathways. Exp Cell Res.

[24] Jin X, Wang J, Xia ZM, Shang CH, Chao QL, Liu YR, Fan HY, Chen DQ, Qiu F, Zhao F (2016). Anti-inflammatory and Anti-oxidative Activities of Paeonol and Its Metabolites Through Blocking MAPK/ERK/p38 Signaling Pathway. Inflammation..

[25] Kim SA, Lee HJ, Ahn KS, Lee EO, Choi SH, Jung SJ, Kim JY, Baek N, Kim SH (2009). Paeonol exerts anti-angiogenic and anti-metastatic activities through downmodulation of Akt activation and inactivation of matrix metalloproteinases. Biol Pharm Bull.

[26] Zhang L, Tao L, Shi T, Zhang F, Sheng X, Cao Y, Zheng S, Wang A, Qian W, Jiang L (2015). Paeonol inhibits B16F10 melanoma metastasis In vitro and In Vivo via disrupting proinflammatory cytokines-mediated NF-kappaB and STAT3 pathways. IUBMB Life.

[27] Yao Z, Shulan Z (2008). Inhibition effect of Guizhi-Fuling-decoction on the invasion of hum an cervical cancer. J Ethnopharmacol.

[28] Li D, Zhang Y, Han H, Geng J, Xie X, Zheng J, Wang Y, Zou X (2012). Effect of lichong decoction on expression of IGF-I and proliferating cell nuclear antigen mRNA in rat model of uterine leiomyoma. J Tradit Chin Med.

[29] Li TF, Lin CC, Tsai HP, Hsu CH, Fu SL (2014). Effects of Kuan-Sin-Yin decoction on immunomodulation and tumorigenesis in mouse tumor models. BMC Complement Altern Med.

[30] Zhou N, Han SY, Zhou F, Li PP (2014). Anti-tumor effect of Shu-Gan-Liang-Xue decoction in breast cancer is related to the inhibition of aromatase and steroid sulfatase expression. J Ethnopharmacol.

[31] Cao Z, Chen X, Lin W, Zhao J, Zheng L, Ye H, Liao L, Du J (2015). Jiedu Xiaozheng Yin decoction inhibits hepatoma cell proliferation by inducing apoptosis via the mitochondrial-mediated pathway. Mol Med Rep.

[32] Zhang QB, Meng XT, Jia QA, Bu Y, Ren ZG, Zhang BH, Tang ZY. Herbal compound Songyou Yin and moderate swimming suppress growth and metastasis of liver cancer by enhancing immune function. Integr Cancer Ther. 2016, in press. 10.1177/1534735415622011 PMC5739186 26699805

[33] Yen GC, Chen YL, Sun FM, Chiang YL, Lu SH, Weng CJ (2011). A comparative study on the effectiveness of cis- and trans-form of cinnamic acid treatments for inhibiting invasive activity of human lung adenocarcinoma cells. Eur J Pharm Sci.

[34] Manikandan P, Murugan RS, Priyadarsini RV, Vinothini G, Nagini S (2010). Eugenol induces apoptosis and inhibits invasion and angiogenesis in a rat model of gastric carcinogenesis induced by MNNG. Life Sci.

[35] Kang HS, Kim J, Lee HJ, Kwon BM, Lee DK, Hong SH (2014). LRP1-dependent pepsin clearance induced by 2’-hydroxycinnamaldehyde attenuates breast cancer cell invasion. Int J Biochem Cell Biol.

[36] Jayasooriya RG, Dilshara MG, Park SR, Choi YH, Hyun JW, Chang WY, Kim GY (2014). 18beta-Glycyrrhetinic acid suppresses TNF-alpha induced matrix metalloproteinase-9 and vascular endothelial growth factor by suppressing the Akt-dependent NF-kappaB pathway. Toxicol In Vitro.

[37] Wang KL, Hsia SM, Chan CJ, Chang FY, Huang CY, Bau DT, Wang PS (2013). Inhibitory effects of isoliquiritigenin on the migration and invasion of human breast cancer cells. Expert Opin Ther Targets.

[38] Kwon GT, Cho HJ, Chung WY, Park KK, Moon A, Park JH (2009). Isoliquiritigenin inhibits migration and invasion of prostate cancer cells: possible mediation by decreased JNK/AP-1 signaling. J Nutr Biochem.

[39] Virtanen SS, Kukkonen-Macchi A, Vainio M, Elima K, Harkonen PL, Jalkanen S, Yegutkin GG (2014). Adenosine inhibits tumor cell invasion via receptor-independent mechanisms. Mol Cancer Res.

[40] Gao Y, Jia Z, Kong X, Li Q, Chang DZ, Wei D, Le X, Suyun H, Huang S, Wang L (2011). Combining betulinic acid and mithramycin a effectively suppresses pancreatic cancer by inhibiting proliferation, invasion, and angiogenesis. Cancer Res.

[41] Liu J, Zheng L, Ma L, Wang B, Zhao Y, Wu N, Liu G, Lin X (2014). Oleanolic acid inhibits proliferation and invasiveness of Kras-transformed cells via autophagy. J Nutr Biochem.

[42] Guo G, Yao W, Zhang Q, Bo Y (2013). Oleanolic acid suppresses migration and invasion of malignant glioma cells by inactivating MAPK/ERK signaling pathway. PLoS One.

[43] Lu JT, He W, Song SS, Wei W (2014). Paeoniflorin inhibited the tumor invasion and metastasis in human hepatocellular carcinoma cells. Bratisl Lek Listy.

[44] Yoshida S, Hirakawa N, Ito K, Miura Y, Yagasaki K (2011). Anti-invasive activity of alpha-tocopherol against hepatoma cells in culture via protein kinase C inhibition. J Clin Biochem Nutr.

[45] Kim SO, Kim MR (2013). [6]-gingerol prevents disassembly of cell junctions and activities of MMPs in invasive human pancreas cancer cells through ERK/NF- kappa B/snail signal transduction pathway. Evid Based Complement Alternat Med.

[46] Weng CJ, Chou CP, Ho CT, Yen GC (2012). Molecular mechanism inhibiting human hepatocarcinoma cell invasion by 6-shogaol and 6-gingerol. Mol Nutr Food Res.

[47] Yagihashi S, Miura Y, Yagasaki K (2008). Inhibitory effect of gingerol on the proliferation and invasion of hepatoma cells in culture. Cytotechnology.

[48] Hsu YL, Hung JY, Tsai YM, Tsai EM, Huang MS, Hou MF, Kuo PL (2015). 6-shogaol, an active constituent of dietary ginger, impairs cancer development and lung metastasis by inhibiting the secretion of CC-chemokine ligand 2 (CCL2) in tumor-associated dendritic cells. J Agric Food Chem.

[49] Ling H, Yang H, Tan SH, Chui WK, Chew EH (2010). 6-Shogaol, an active constituent of ginger, inhibits breast cancer cell invasion by reducing matrix metalloproteinase-9 expression via blockade of nuclear factor-kappaB activation. Br J Pharmacol.

[50] Weng CJ, Wu CF, Huang HW, Ho CT, Yen GC (2010). Anti-invasion effects of 6-shogaol and 6-gingerol, two active components in ginger, on human hepatocarcinoma cells. Mol Nutr Food Res.

[51] Friedl P, Alexander S (2011). Cancer invasion and the microenvironment: plasticity and reciprocity. Cell.

[52] Dhillon AS, Hagan S, Rath O, Kolch W (2007). MAP kinase signalling pathways in cancer. Oncogene.

[53] Hu H, Li Z, Zhu X, Lin R, Peng J, Tao J, Chen L (2013). GuaLou GuiZhi decoction inhibits LPS-induced microglial cell motility through the MAPK signaling pathway. Int J Mol Med.

[54] Koul HK, Pal M, Koul S (2013). Role of p38 MAP kinase signal transduction in solid tumors. Genes Cancer.

[55] Chen Y, Gao C, Ma Y, Qiu F (2013). Pharmacokinetic study of multiple active constituents after oral gavage of Guizhi decoction in rats using a LC-MS/MS method. Eur J Drug Metab Pharmacokinet.

[56] Wang K, Karin M (2015). Tumor-elicited inflammation and colorectal cancer. Adv Cancer Res.

[57] Solinas G, Marchesi F, Garlanda C, Mantovani A, Allavena P (2010). Inflammation-mediated promotion of invasion and metastasis. Cancer Metastasis Rev.

