# Supplementary material for: Geijigajakyak decoction inhibits the motility and tumorigenesis of colorectal cancer cells
Source: BMC Complement Altern Med. 2016 Aug 15;16:288. doi: 10.1186/s12906-016-1281-z (PMC4986256; doi:10.1186/s12906-016-1281-z)
Supplement: Additional file 1: Table S1. — Herbal information of five components of GJD used in this study. (DOCX 15 kb) [file 12906_2016_1281_MOESM1_ESM.docx]

**Additional file 1: Table S1** Herbal information of five components of GJD used in this study

| Herb | Distribution Company  (serial number) | [Manufacturing](javascript:endicAutoLink('manufacturing');) [Company](javascript:endicAutoLink('company');) | Inspection Agency  (examination date) |
| --- | --- | --- | --- |
| Cinnamomi Ramulus | Omniherb, Daegu, Korea  <http://www.omniherb.com/>  +82-80-345-1255  (D1304144V) | Gioherb, Seoul, Korea  <http://www.gioherb.com/>  +82-2-963-4628 | Dongwoodang  (2013.05.06) |
| Glycyrrhizae Radix | Omniherb, Daegu, Korea  <http://www.omniherb.com/>  +82-80-345-1255  (D1307117C) | Dongwoodang, Yeongcheon, Korea  <http://www.dongwoodang.co.kr/>  +82-54-333-8084 | Dongwoodang  (2013.07.31) |
| Paeoniae Radix | Omniherb, Daegu, Korea  <http://www.omniherb.com/>  +82-80-345-1255  (D1302166K) | Dongwoodang, Yeongcheon, Korea  <http://www.dongwoodang.co.kr/>  +82-54-333-8084 | Dongwoodang  (2013.02.28) |
| Zingiberis Rhizoma | Omniherb, Daegu, Korea  <http://www.omniherb.com/>  +82-80-345-1255  (D13035143K) | Dongwoodang, Yeongcheon, Korea  <http://www.dongwoodang.co.kr/>  +82-54-333-8084 | Dongwoodang  (2013.04.06) |
| Ziziphi Fructus | Omniherb, Daegu, Korea  <http://www.omniherb.com/>  +82-80-345-1255  (D1310190K) | Gunwidaechu  926-7, Wansandong, Yeongcheon, Gyeongbuk, Korea  +82-11-9374-459* | Gunwidaechu  (2013.11.01) |

These information are from labels attached to the products, which were purchased for this study. In Korea, all companies dealing with herbs are legal [pharmaceutical](javascript:endicAutoLink('pharmaceutical');) companies. To maintain the high quality of their herbal material, they perform qualifying examination of obtained herbs via many types of pharmacological and [pharmaceutical](javascript:endicAutoLink('pharmaceutical');) tests including constituents, toxicities, pesticide residue of herbs. After passing these Q/C test, they distribute their herbs to oriental clinic, medical clinic, and research institution, etc.
